# Editorial: Chemical composition and antimicrobial activity of essential oils

**DOI:** 10.3389/fphar.2023.1120756

**Published:** 2023-01-13

**Authors:** Mijat Božović, Milan Mladenović, Rino Ragno

**Affiliations:** ^1^ Department of Biology, Faculty of Science and Mathematics, University of Montenegro, Podgorica, Montenegro; ^2^ Department of Chemistry, Faculty of Science, University of Kragujevac, Kragujevac, Serbia; ^3^ Department of Drug Chemistry and Technology, Faculty of Pharmacy and Medicine, Sapienza University of Rome, Rome, Lazio, Italy

**Keywords:** essential oil, antimicrobial activity, chemical composition, antiviral activity, endemic plants

Since the increased prevalence of drug-resistant pathogens aggravates the successful treatment of microbial diseases, exploration of new remedies is a major and very important challenge of research nowadays. In this context, essential oils (EOs) have received a great deal of scientific attention, because of both potential antimicrobial activities and reduced side effects. The aim of this Research Topic was to bring together original research and review papers, providing relevant information on the development of extraction methods, chemical characterization strategies and the analysis of antimicrobial activity of EOs and/or their chemical constituents. In this editorial, we summarize the main findings and perspectives of each of the published articles.

Ćavar Zeljković et al. reported a novel *in vitro* antiviral activity study of 19 EOs extracted from Lamiaceae species. The study also included the main monoterpene constituents of those EOs, and the analysis was conducted against severe acute respiratory syndrome coronavirus 2 (SARS-CoV-2). The results obtained in this study show that the essential oil (EO) of *Mentha* x *villosa* Huds. possessed the greatest antiviral activity, whereas the EOs of *Ziziphora clinopodioides* Lam. and *Micromeria thymifolia* (Scop.) Fritsch were characterized by the lowest potencies. Additionally, the monoterpenes carvone and carvacrol were found to exhibit significant activity. These findings represent a significant contribution to the antiviral activity of EOs and can be used in the development of various measures against SARS-CoV-2, which is associated with the current global pandemic.

Plants belonging to the Lamiaceae family are well known for their EO constituents, many of which are an important source of antibacterial and antifungal agents and are therefore part of traditional and modern medical treatment. However, many species are still unexplored, and there is great interest in new bioactive molecules. Based on numerous scientific evidences of different *Thymus* species and their EOs against various respiratory disorders, Rehman et al. analyzed the EO of *Th. serrulatus* Hochst. ex Benth, an Ethiopian endemic plant which is traditionally used for various disease conditions (e.g., flue and cough). This study included the chemical composition analysis, assessment of the possible tracheal relaxant effects in an *ex vivo* model, along with antimicrobial properties. The EO was found to be active against strains involved in respiratory infections, particularly *Staphylococcus aureus* and *Candida albicans*. It also caused tracheal relaxation that was probably mediated by an anticholinergic effect, Ca^++^ channel blockade and phosphodiesterase inhibition, as concluded by the authors.

Furthermore, the first report on the chemical composition, anti-*Candida* and antibacterial activities of the EO of *Adenophyllum porophyllum* var. *cancellatum* (Cass.) Strother was given by Aguilar-Rodríguez et al. This aromatic annual plant belongs to the Asteraceae family and it is used in traditional medicine in Mexico to treat numerous skin problems, such as irritation or infections, but also ulcers and wounds. The results presented in this study indicate the great potential of this plant as an alternative antimicrobial agent that can be used to infectious diseases control, especially against *Candida* species that have been found to be the most susceptible. Trans- and cis-pinocamphone, limonene and pinocarvone are reported as the main constituents of the EO.

The antimicrobial activity of *Eugenia brejoensis* Mazine EO in a murine model of skin infection caused by *S. aureus* was reported by Diniz et al. This is an endemic Myrtaceae species from the semi-arid Caatinga domain in northeastern Brazil. The results show the positive effects of this EO on wound contraction associated with a significant reduction in bacterial load, as well as the release of inflammatory mediators (IL-6, IL-17A, TNF-α, NO, VEGF). These data provide new insights into the anti-infective potential, anti-inflammatory efficacy and healing properties, making this EO an interesting option for drug development.

1,8-cineole (eucalyptol) is well-known as the major bacteriostatic agent in the EO of *Eucalyptus globulus* Labill. (Myrtaceae). In addition, this oxygenated monoterpene possesses remarkable anti-inflammatory properties. After being incapsulated, the molecular mechanisms of its effects on the growth performance and intestinal microbiota under heat stress have been investigated and reported by Jiang et al. This study demonstrates that 1,8-cineole could prevent high temperature-induced intestinal flora disorders and inflammation, by increasing the abundance of *Lactobacillus* and preventing *Salmonella* expression. Taking into account that intestinal dysbiosis is frequently associated with inflammatory diseases, the ability of 1,8-cineole to prevent these disorders makes it an alternative dietary supplement.

Finally, the comprehensiveness of this Research Topic is enhanced by Bunse et al. who summarize EOs’ potential as multicomponent mixtures for human health and wellbeing in a review paper. The main emphasis was set on antibacterial, antiviral and anti-inflammatory properties that qualify EOs for both causal and symptomatic therapy of numerous diseases, as well as for prevention. The authors point out that the variety of molecules in EOs with different functionalities exhibits a broad array of chemical and physical properties. This diversity is responsible for the well-known multi-target activity of EOs, in contrast to their isolated, individual compounds. EO ingredients can complement or potentiate each other through the mechanisms of synergistic and/or antagonistic actions, often neutralizing toxic effects. These compounds also interact with their environment influencing the activity or conformation of molecular targets. Numerous pharmacological effects and therapeutic uses of EOs are based on their ability to bind receptors and trigger physiological and/or psychological responses. The authors particularly emphasized the positive influence of EOs in the successful treatment of infections, especially those caused by multidrug-resistant bacteria. Moreover, studies have shown that these volatile mixtures in combination with antibiotics have capacity to enhance the antibiotic effect. This is of particular interest due to possibility to minimize or even prevent the careless use of antibiotics nowadays.

Considering the current importance of the discovery of new antimicrobials, in terms of growing microbial resistance, the contributions to this Research Topic represent significant data and can be used in the development of some new drug formulations. It is of particular interest to mention the antiviral activity against SARS-CoV-2 as a recent global problem, as well as some new data on antimicrobial activity related to the endemic *Th. serrulatus* Hochst. ex Benth. and *E. brejoensis* Mazine. Although often representing vulnerable plant sources of national or regional importance, endemic species can also offer some new bioactive molecules and these data are always of particular scientific importance. However, limitations should always be considered in terms of reasonable harvesting and use of natural resources. Scientific confirmations of plant species that are traditionally used in certain regions are of great value and are always a good way to access various pharmacological activities and benefits. In this sense, the data obtained on the Mexican *A. porophyllum* var. *cancellatum* (Cass.) Strother are appreciable. Taking into account all the results and contributions, further and broader analyzes can be suggested.

